# Oncological emergency surgery for metachronous large and small bowel metastases after pancreaticoduodenectomy for pancreatic cancer: a case report

**DOI:** 10.1186/s40792-018-0506-4

**Published:** 2018-08-22

**Authors:** Mamoru Miyasaka, Takehiro Noji, Kimitaka Tanaka, Yoshitsugu Nakanishi, Toshimichi Asano, Yuma Ebihara, Yo Kurashima, Toru Nakamura, Soichi Murakami, Takahiro Tsuchikawa, Keisuke Okamura, Toshiaki Shichinohe, Satoshi Hirano

**Affiliations:** Department of Gastroenterological Surgery II, Hokkaido University Faculty of Medicine, Kita 15 Nishi 7, Kita-ku, Sapporo, Hokkaido 060-8638 Japan

**Keywords:** Metachronous intestinal metastases, Pancreatic cancer, Oncological emergency, Pancreatoduodenectomy

## Abstract

**Background:**

A surgical case of metachronous metastases of pancreatic head cancer (PC) to the large and small bowel is extremely rare. Therefore, there are only a few reports about surgery for intestinal metastases from PC. An oncologic emergency is defined as an acute, potentially life-threatening condition in a cancer patient that developed directly or indirectly because of the malignant disease or cancer treatment.

**Case presentation:**

A 63-year-old man with PC underwent pancreaticoduodenectomy after receiving neoadjuvant chemotherapy with gemcitabine and S-1. Histopathologically, the tumor was diagnosed as poorly differentiated, tubular adenocarcinoma, with pT2, N0, pStage IB according to the UICC classification, seventh edition. R0 was achieved. Three months after pancreatoduodenectomy, blood tests showed coagulation derangements with high C-reactive protein (CRP 11.30 mg/dl). Computed tomography (CT) scan revealed a 55-mm mass alongside the transverse colon. During 2 weeks of follow-up, the coagulation derangement and elevated CRP persisted. Repeat CT showed that the tumor enlarged to 65 mm, and an additional mass, 25 mm in diameter, was detected in the jejunum. He was hospitalized due to abdominal pain and diarrhea with persistent high fever and was inspected; however, there was no evidence for infections. With the understanding that his life-threatening symptoms were secondary to the underlying malignancy, extirpation of the tumors combined with partial resection of the transverse colon and the jejunum was performed on the eighth day of hospitalization, on an emergency basis.

The lesions were identified as large and small bowel metastases from PC because histopathological examination revealed morphological features similar to the primary disease.

Immediately after the emergency surgery, the fever resolved and the CRP level normalized. He was discharged and received nab-paclitaxel with gemcitabine chemotherapy for 2 months postoperatively. He selected for best supportive care after this. The patient died due to a relapse with mesenteric lymph node metastasis 7 months after the emergency surgery.

**Conclusion:**

Surgery as an oncological emergency for selected patients could sometimes contribute to improving patient’s quality of life.

## Background

Recurrences after curative resection for pancreatic cancer (PC) are commonly found in the liver with locoregional recurrence being the second most frequent site [1]. As recurrence is associated with a significantly poorer prognosis, surgical resection of the recurrence site has generally been unacceptable. A surgical case of metachronous metastases of PC to the large and small bowel is extremely rare [1]. Therefore, there are only a few reports about surgery for intestinal metastases from PC [2–4].

An oncologic emergency is defined as an acute, potentially life-threatening condition in a cancer patient that developed directly or indirectly because of the malignant disease or cancer treatment. Even in terminal cancer patients, there were several reports about the treatment with surgical intervention for oncological emergencies [5].

Herein, we present a case involving pancreatic head cancer requiring surgery for an oncological emergency relating to metachronous intestinal metastatic lesions after pancreatoduodenectomy (PD).

## Case presentation

A 63-year-old man with pancreatic head cancer underwent pancreaticoduodenectomy (PD) after receiving two courses of neoadjuvant chemotherapy with gemcitabine (GEM) and S-1 for 6 months preoperatively based on the protocol of clinical research (Japan Adjuvant Study Group of Pancreatic Cancer 04 study). Ascites cytology was negative of cancer cell. Histopathologically, the tumor was diagnosed as poorly differentiated, tubular adenocarcinoma, with pT2, N0, pStage IB according to the UICC classification, seventh edition [6]. R0 was achieved.

**Fig. 1:**
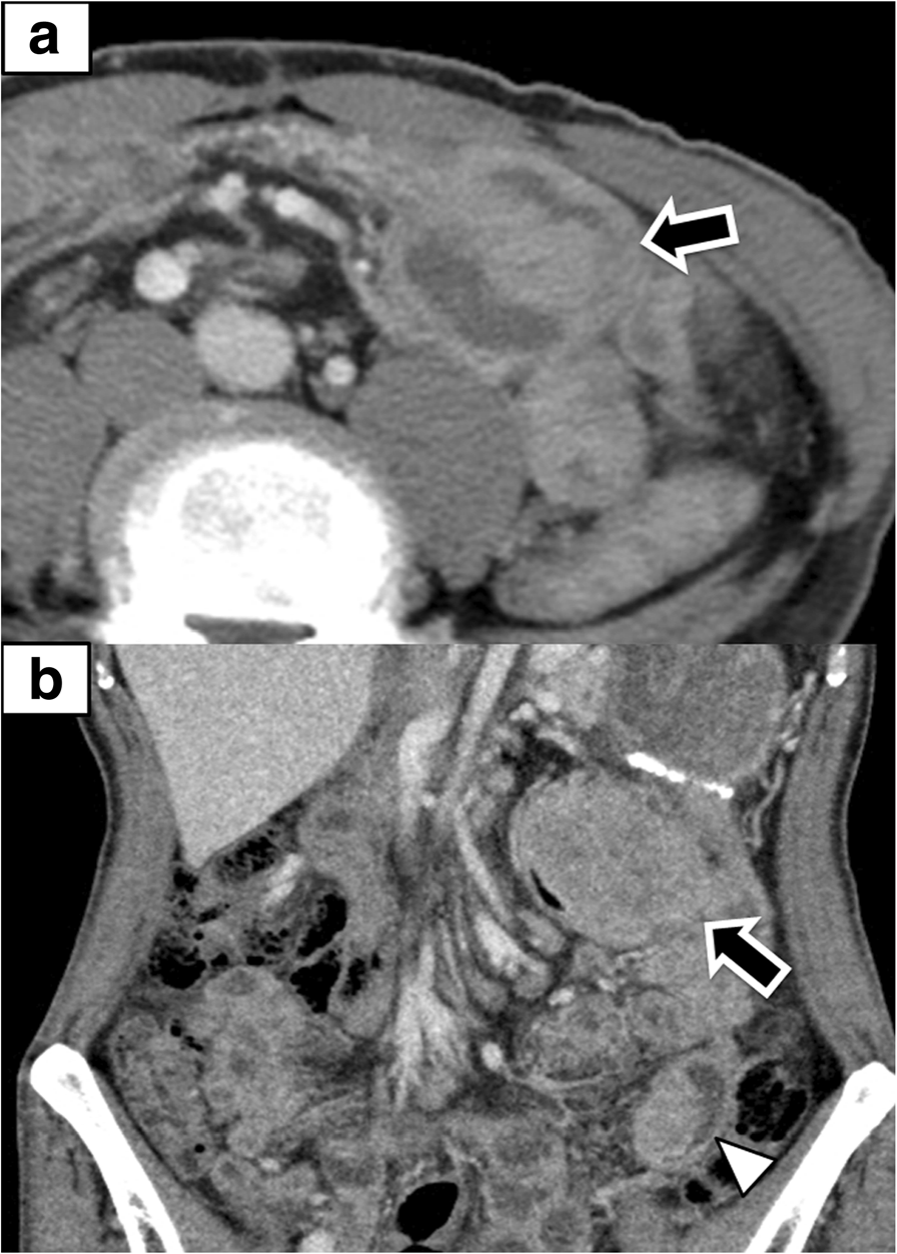
**a** Contrast-enhanced CT showing a mass, 55 mm in diameter, alongside the transverse colon. **b** Contrast-enhanced CT showing that the mass alongside the transverse colon enlarged to 65 mm, and an additional mass, 25 mm in diameter, was found in the jejunum. Black arrow—the lesion alongside the transverse colon; white triangle—the jejunal lesion

**Fig. 2:**
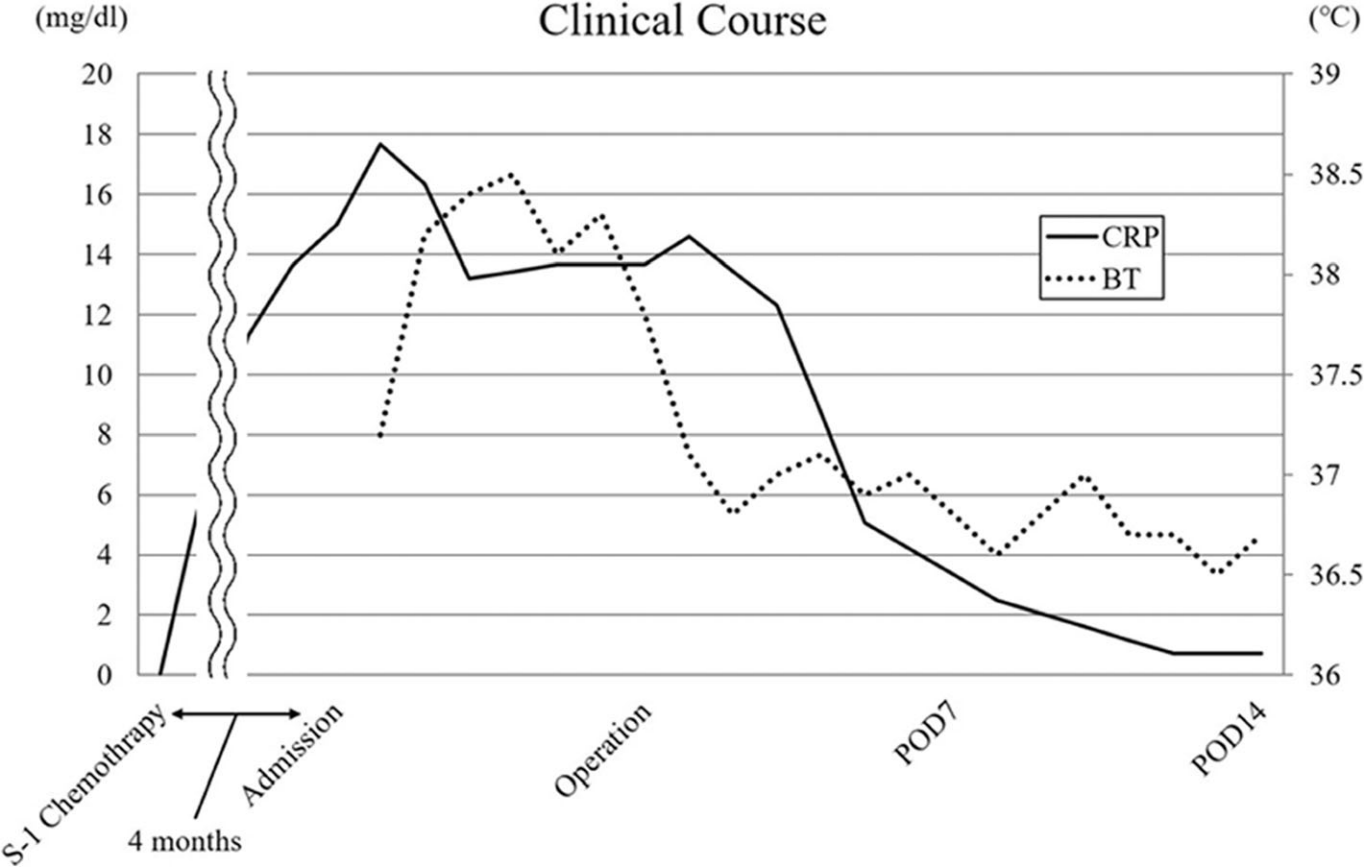
Clinical course after surgery. Body BT, body temperature; CRP, C-reactive protein; POD, postoperative day. During 2 weeks of follow-up, the coagulation derangement and elevated CRP (17.66 mg/dl) persisted. After the emergency surgery, the fever resolved and the CRP level normalized

After the surgery, he received adjuvant S-1 therapy. Three months after PD, blood tests showed coagulation derangements with high C-reactive protein (CRP 11.30 mg/dl). Computed tomography scan (CT) revealed a 55-mm mass alongside the transverse colon (Fig. 1a). During 2 weeks of follow-up, the coagulation derangement and elevated CRP (17.66 mg/dl) persisted (Fig. 2). Repeat CT showed that the tumor enlarged to 65 mm, and an additional mass, 25 mm in diameter, was detected in the jejunum (Fig. 1b). He was hospitalized due to abdominal pain and diarrhea with persistent high fever and was inspected; however, there was no evidence for infections. With the understanding that his life-threatening symptoms were secondary to the underlying malignancy, extirpation of the tumors combined with partial resection of the transverse colon and the jejunum was performed on the eighth day of hospitalization, on an emergency basis.

**Fig. 3:**
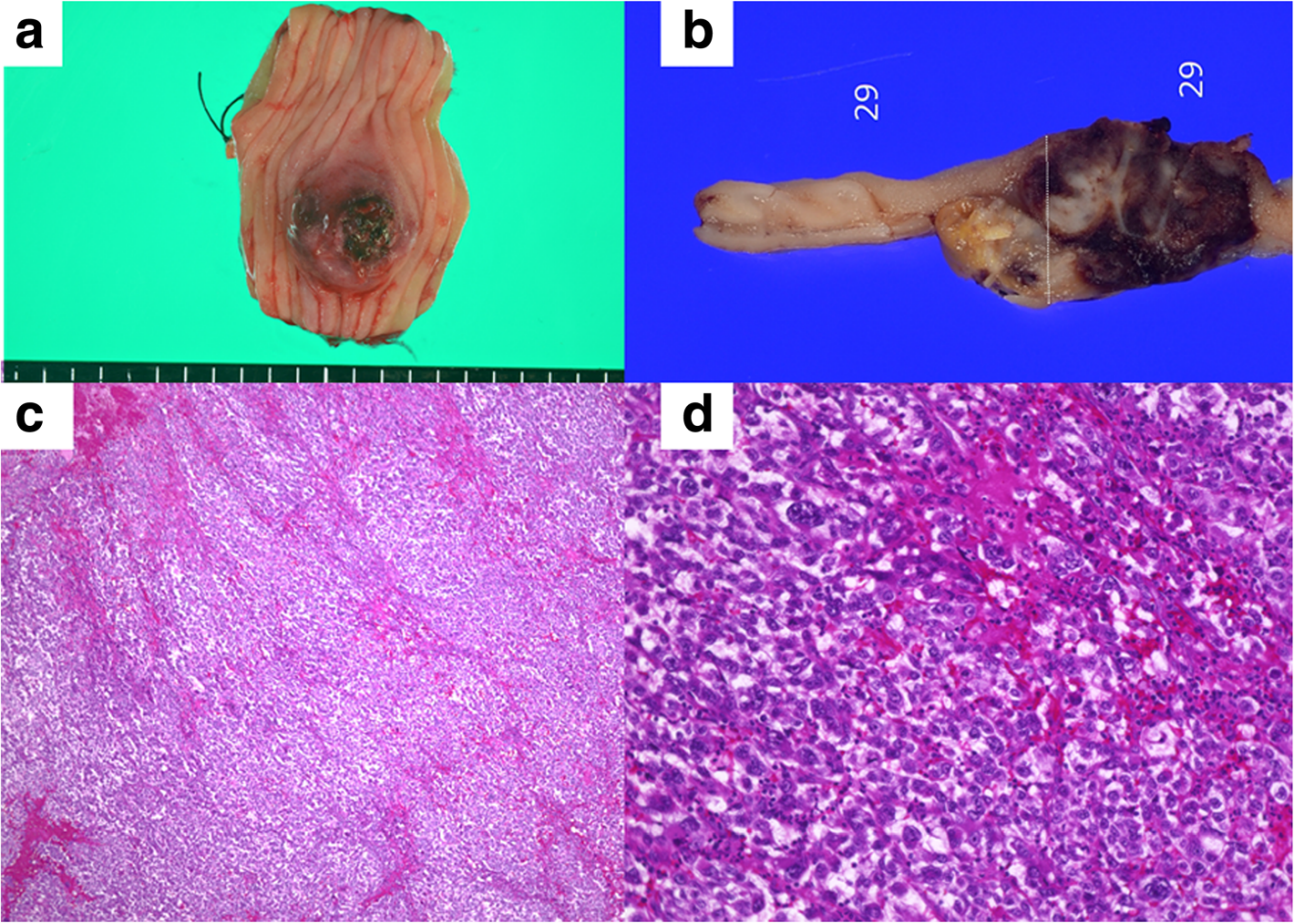
Histopathological findings of the jejunum. **a** The resected specimen of the jejunum had a submucosal tumor 20 mm in diameter. **b** There was no obvious exposure of the tumor to the mucosal and serosal surface. **c** Hematoxylin-eosin stain × 20. **d** Hematoxylin-eosin stain × 400. The proliferation of the tumor cells with high nucleo to cytoplasmic ratio and poor binding. The morphological features were similar to the resected pancreatic cancer

**Fig. 4:**
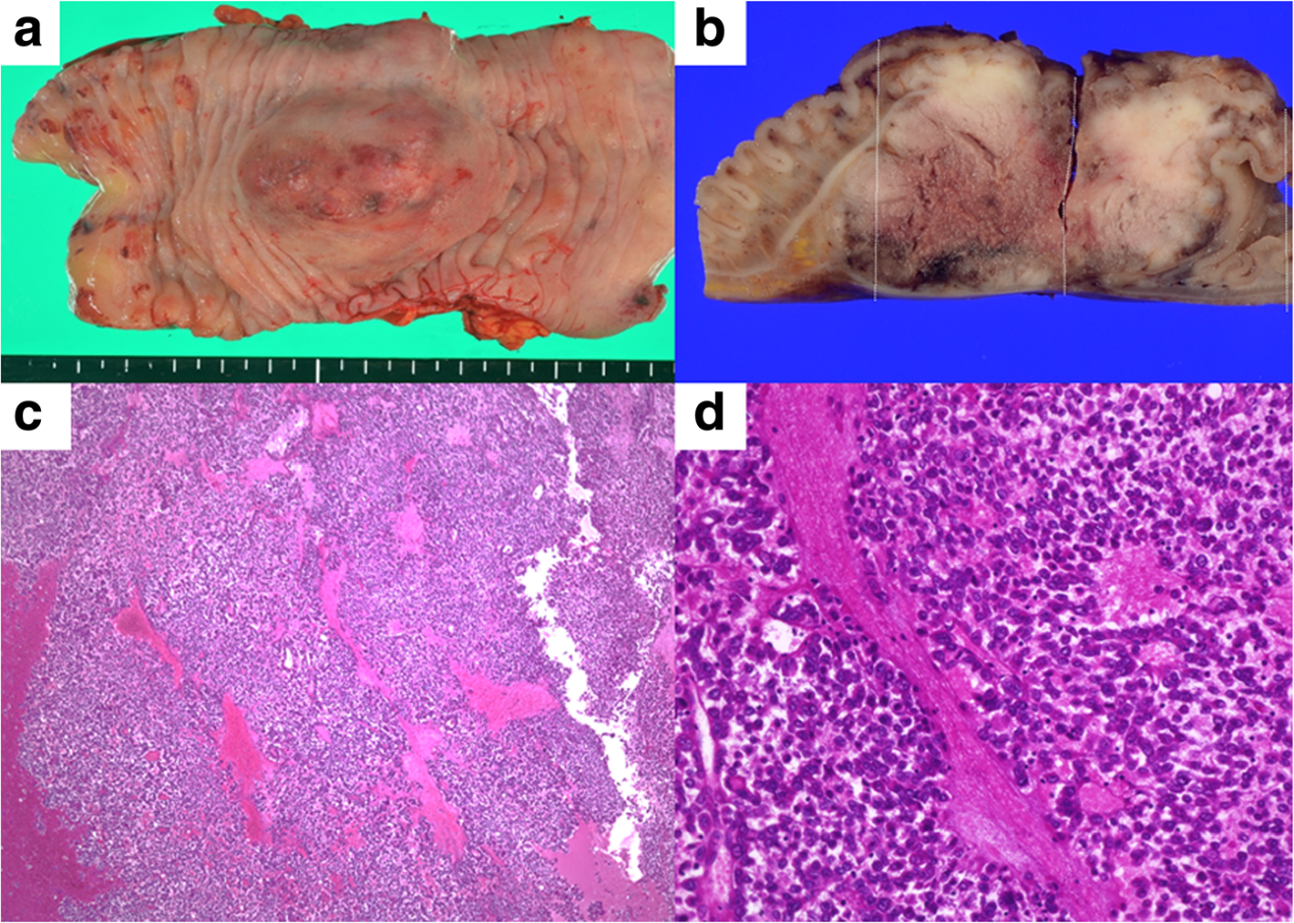
Histopathological findings of the transverse colon. **a** The resected specimen of the transverse colon had a submucosal tumor 60 mm in diameter. **b** There was no obvious exposure of the tumor to the mucosal and serosal surface. **c** Hematoxylin-eosin stain × 20. **d** Hematoxylin-eosin stain × 400. The proliferation of the tumor cells with high nucleo to cytoplasmic ratio and poor binding. The morphological features were similar to the resected pancreatic cancer

Figures 3 and 4 showed the histopathological findings of the two lesions. In both resected specimens, the tumor extended to the submucosal layer and occluded the lumen of the bowels. The lesions were identified as large and small bowel metastases from PC because histopathological examination revealed morphological features similar to the primary disease. Ascites cytology at this time was also negative of cancer.

Immediately after the emergency surgery, the fever resolved and the CRP level normalized (Fig. 2). He was discharged and received nab-paclitaxel with GEM chemotherapy for 2 months postoperatively. He selected for best supportive care after this. The patient died due to a relapse with mesenteric lymph node metastasis 7 months after the emergency surgery (13 months after the first surgery).

### Discussion

In general, patients with PC recurrence are not considered as surgical candidates. Even if a metastatic lesion was thought to be solitary, it is usually treated as a systemic disease. However, in oncological emergencies, surgical intervention should be considered. In the past decades, several reviews about oncological emergencies and their general management have been published [7]. Obstruction of the gastrointestinal tract is the most frequent surgical emergency seen in practice [8] [9]. In malignancies, surgical interventions such as bowel resection, bypass, or ileostomy may provide good palliation by reducing symptoms and alleviating obstruction but remains dependent on the disease extension in each individual patient [9].

As the patient’s condition is often very poor in the emergency setting, especially for patients with end-stage disease, emergency surgery is associated with rates of morbidity of up to 61%, 30-day mortality of 9.8%, and overall mortality of 15–37% [5]. On the other hand, endoscopic alternatives for surgery include tumor ablation and decompression by stent placement [10]. Thus, in case of malignant obstruction, some reports highlighted that surgery for malignant obstruction should be reserved for patients with resectable disease, good performance status (ECOG > 1), and a life expectancy of more than 6 months [8].

In the present case, the patient had deranged coagulation, fever, and elevated CRP, and in the absence of infection or others diagnoses, malignancy was presumed to be the cause. Tumor-produced inflammatory cytokines sometimes cause fever, which is commonly seen in hematologic disease, but also occurs in solid tumors. Thus, the surgical intervention was performed on an emergency basis. Furthermore, as there were two lesions in the intestine, bowel resection was performed instead of a bypass, which was less likely to improve the bowel obstruction. Consequently, he was able to go back home and survived for 7 months after the emergency surgery.

Because there were few reports about metachronous intestinal metastases after PC surgery (Table 1), it is unclear whether surgery could improve these patients’ prognoses. However, in oncological emergencies, surgical intervention is expected to improve patient’s quality of life. Although the patient was diagnosed in a terminal cancer stage, there might be an advantage for resecting metastatic tumors. Based on the advancement of multimodality treatments, such as chemotherapy, in the recent years, even metastatic lesions from PC would turn to be resectable in some cases. Therefore, surgical resection should be considered for large and small bowel metastasis from resected PC in selected patients.

**Table 1:** Reports of surgical treatment for intestinal metastasis from resected PC

Author and year of publication	Age/sex	Primary PC site/TNM stage*	AC after pancreatectomy	DFI (months)	Symptoms	Metastatic site	AC after metasectomy	TI (months)
Ogu US, 2012^4**^	85/F	Ph/T3N0M0/IIA	NA	24	Abdominal pain	Sigmoid colon	NA	NA
Inada K, 2013^3**^	62/M	Ph/T2N1M0/IIB	GEM	86	Abdominal pain	Ascending colon	NA	14
Kim W, 2015^2**^	64/M	Pb/T3N0M0/IIA	None	23	Abdominal pain	Cecum	GEM	6
Our case, 2018	63/M	Ph/T2N0M0/IB	S-1	4	Fever Bowel obstruction	Transverse colon Jejunum	GEM nab-PTX	7

*AC* adjuvant chemotherapy; *DFI* disease-free interval; *F* female; *GEM* gemcitabine; *NA* not available; *nab-PTX* nab-paclitaxel; *M* male; *PC* pancreatic cancer; *Pb* pancreatic body; *Ph* pancreatic head; *S-1* tegafur, gimeracil, and oteracil potassium; *TI* months between the date of metasectomy and date of the death or last follow-up, whichever occurred first

*According to the UICC classification seventh edition; ** reference number

## Conclusions

We report a rare case of metachronous intestinal metastases from resected PC. Surgery as an oncological emergency for selected patients could sometimes contribute to improving patient’s quality of life.

## Abbreviations

CRP: C-reactive protein
CT: Computed tomography scan
GEM: Gemcitabine
PC: Pancreatic cancer
PD: Pancreatoduodenectomy
